# Attenuation and molecular characterization of fowl adenovirus 8b propagated in a bioreactor and its immunogenicity, efficacy, and virus shedding in broiler chickens

**DOI:** 10.14202/vetworld.2024.744-755

**Published:** 2024-04-03

**Authors:** Chidozie C. Ugwu, Mohd Hair-Bejo, Mat I. Nurulfiza, Abdul R. Omar, Aini Ideris

**Affiliations:** 1 Department of Veterinary Pathology and Microbiology Faculty of Veterinary Medicine, Universiti Putra Malaysia, Serdang 43400, Selangor, Malaysia; 2 Department of Animal Science and Technology, Federal University of Technology, Owerri 460114, Imo State, Nigeria; 3 Laboratory of Vaccine and Biomolecules, Institute of Bioscience, Universiti Putra Malaysia, Serdang 43400, Selangor, Malaysia; 4 Department of Cell and Molecular Biology, Faculty of Biotechnology and Biomolecular Sciences, Universiti Putra Malaysia, Serdang 43400, Selangor, Malaysia

**Keywords:** antibodies, attenuation, fowl adenovirus 8b, T lymphocytes, vaccines, virus shedding

## Abstract

**Background and Aim:**

Live-attenuated vaccines are the most successful type of vaccine and could be useful in controlling fowl adenovirus (FAdV) 8b infection. This study aimed to attenuate, molecularly characterize, and determine the immunogenicity, efficacy, and challenge virus shedding in broiler chickens.

**Materials and Methods:**

The FAdV 8b isolate (UPM08136) was passaged onto chicken embryo liver (CEL) cells until attenuation. We sequenced and analyzed the hexon and fiber genes of the passage isolates. The attenuated bioreactor-passage isolate was inoculated into 1-day-old broiler chickens with (attenuated and inactivated) and without booster groups and challenged. Body weight (BW), liver weight (LW), liver: body weight ratio (LBR), FAdV antibody titers, T-lymphocyte subpopulation in the liver, spleen, and thymus, and challenge virus load and shedding were measured.

**Results:**

Typical cytopathic effects with novel genetic changes on CEL cells were observed. The uninoculated control-challenged (UCC) group had significantly lower BW and higher LW and LBR than the inoculated groups. A significantly higher FAdV antibody titer was observed in the challenged non-booster and attenuated booster groups than in the UCC group. T cells in the spleen and thymus of the liver of inoculated chickens were higher than uninoculated control group levels at all-time points and at different times. A significantly higher FAdV challenge virus load was observed in the liver and shedding in the cloaca of UCC chickens than in non-booster chickens.

**Conclusion:**

The FAdV 8b isolate was successfully attenuated, safe, and immunogenic. It reduces virus shedding and is effective and recommended as a vaccine against FAdV infection in broiler chickens.

## Introduction

Fowl adenovirus (FAdV) serotype 8b belongs to Group E of the genus aviadenovirus in the family *Adenoviridae* [1, 2] and is an etiologic agent of inclusion body hepatitis (IBH) in broiler chickens that are 3–7 weeks of age and cause mortality of 2%–30% [3–5]. IBH pathogenesis may involve sudden death with no obvious clinical signs [6]. Surviving chickens may have pale comb and wattle, insensitivity, abnormal position, occasional nervous signs, and reduced growth [5]. Lesions that are frequently observed include pale, friable, hemorrhagic liver, hemorrhages in the thigh and kidney, and congestion in the ventriculus and spleen [6]. Histopathological changes may include basophilic and/or eosinophilic intranuclear inclusion bodies in the liver and pancreas, inflammatory infiltration in organs [5, 7], and lymphoid depletion in the thymus, bursa of Fabricius, and spleen [8].

In view of the short course of IBH, prevention through vaccination is an ideal control measure [3]. In addition, vaccinating the parent stock with a live-attenuated FAdV vaccine may help to limit vertical transmission and provide passive immunity, which is beneficial for the protection of progenies. Live-attenuated vaccines are believed to be superior to other vaccines because of their superior ability to induce higher humoral and cell-mediated immunity [9, 10]. They mimic natural infection, which explains their superior immunogenic competency, and they also offer better administration methods; however, they may revert to virulence [11]. Attenuated vaccines are produced if the virus is weakened so that it is incapable of causing disease but is still immunogenic. However, to overcome the problem of reversion to virulence, molecular monitoring is the gold standard [11]. Such molecular characterization of FAdV 8b passage in chicken embryonated eggs (CEE) revealed changes in the L1 loop region of the hexon and knob region of the fiber genes [12].

The absence of cross-protection is an important epidemiological feature of IBH that requires the development of a serotype-specific vaccine [6]. However, the efficacy of vaccines against viral diseases depends on their ability to induce a sufficient amount of cellular immunity, particularly cytotoxic T cells [13]. Unfortunately, there is little information on the cellular immune response of chickens to attenuated FAdV 8b. However, live-attenuated FAdV 4 vaccines provide full protection when administered to day-old chicks [13]. Attenuated FAdV protects against T-cell and B-cell depletion and is effective in chicks with low antibody production [14], suggesting other protective pathways. Therefore, there is a lack of knowledge on how to examine the cellular immune response of chickens to attenuated FAdV 8b.

We hypothesized that attenuated FAdV 8b may be useful in the prevention and control of IBH. This study was conducted to determine the pathogenicity, humoral and cellular immunogenicity, efficacy, and virus shedding of attenuated FAdV 8b adapted to bioreactor propagation.

## Materials and Methods

### Ethical approval

This study was approved by the Universiti Putra Malaysia Institutional Animal Care and Use Committee (IACUC) in a letter with ref number; UPM/IACUC/AUP-R086/2018, dated March 08, 2019) according to the guidelines of the Declaration of Helsinki.

### Study period and location

This study was carried out from March 2019 to February 2021 at the Virology Laboratory, Department of Veterinary Pathology and Microbiology, Fakulti Perubatan Veterinar, Universiti Putra Malaysia.

### Virus

The FAdV serotype 8b isolate UPM08136EEP1 was propagated once in specific pathogen-free (SPF) CEE and stored in the Virology Laboratory of Fakulti Perubatan Veterinar, Universiti Putra Malaysia. The isolate was filtered with a 0.45-μm syringe filter (Corning, Glendale, Arizona, USA) before use.

### FAdV attenuation in prepared chicken embryo liver (CEL) cells

Aseptically harvested liver tissue from 15-day-old SPF CEE rats was used to prepare CEL cells as previously described [15]. Confluent CEL cells in tissue culture (TC) flasks were used to passage UPM08136CELP0 20 times to yield CELP1–CELP20, and molecular analysis was performed every five passages. TC flasks with viral culture were observed daily with the aid of an inverted light microscope (Leica Microsystems, Germany) for cytopathic effect (CPE) until 70% CPE was achieved, whereas control flasks were also observed daily. The CPE photo for each passage and the corresponding control was captured using an inverse microscope (Nikon Eclipse TS100, Japan) with the aid of the NIS elements imaging system (Nikon Digital sight DS-U2, Japan) powered by a PowerLogic computer with a 17′′ monitor (Lenovo, China). The CELP20 isolate, which exhibited delayed CPE with molecular changes, was propagated 1 in CEL cells adapted to Cytodex™ 1 microcarrier (GE Healthcare, Uppsala, Sweden) in a stirred tank bioreactor to produce UPM08136P20B1 without any molecular changes in hexon and fiber genes and with a titer of 106.5 TCID50 [16].

### Molecular characterization of the hexon and fiber genes

The innuPREP virus DNA kit (AnalytikJena, Berlin, Germany) was used to extract DNA from the SPF egg isolate and CEL cell passage isolates 1, 5, 10, 15, 20, and bioreactor isolate P20B1 from the UPM08136 isolate. DNA concentration was measured at a dilution factor of 70 using an ultraviolet–visible spectrophotometer (UV-1601, PC, Shimadzu, Japan). HexA1/HexB1 [17] and fbrF/fbrR primers [16] were used to amplify hexon and fiber genes, respectively. Assembling consensus sequences and amino acid deduction were performed using BioEdit software v 7.2.5 (https://bioedit.software.informer.com/7.2/) and ExPasy software (www.expasy.ch/tools/dna.html), respectively [16]. Sequences were deposited in GenBank using NCBI Bankit tool (https://www.ncbi.nlm.nih.gov/WebSub/), and accession numbers were obtained.

### Inoculum and viral challenge

FAdV 8b (UPM08136P20) was propagated once in a stirred tank bioreactor using CEL cells adapted to Cytodex™ 1 microcarriers to produce UPM08136P20B1 [16], whereas inactivated UPM08136P5B1 was prepared as previously reported [18] and used as attenuated and inactivated inocula, respectively, in the experimental animal trial. UPM11142CELP5 isolates passaged 2× in SPF CEE to obtain UPM11142P5EP2 with a titer of 10^8^ egg Infective dose (EID)50/mL were filtered and used as a challenge virus for the chicken trial.

### Pathogenicity, immunogenicity, safety, and efficacy of the UPM08136CEL20B1 inactivated FAdV serotype 8b isolate on commercial broiler chickens

A total of 116 commercial broiler chickens aged 1 day old without any disease and unvaccinated were randomly assigned into Groups A and B and subgroups A1, B1, B2, and B3 in such a way that four chickens were sampled from each group on the sampling days, as described in Table-1. The chickens were housed in cages. Feed and water were provided ad libitum, and clinical signs were monitored daily. Group B chickens were subcutaneously inoculated with 0.5 mL of UPM08136CEL20B1 (10^6.5^) tissue culture infective dose (TCID50)/mL at day 0, whereas Group A chickens were not. Booster doses were administered to Group B2 (live-attenuated UPM08136P20B1) and Group B3 (inactivated UPM08136P5B1) at 14-day post-inoculation (dpi). On day 28 pi, 0.5 mL of UPM11142CEL5EP2 (10^8^ TCID50/mL) was administered intramuscularly as a challenge to eight chickens in each group. Sampling was performed as previously described by Ugwu et al. [18].

**Table-1:** Design for pathogenicity, immunogenicity, and efficacy of attenuated UPM08136CELP20B1 on commercial broiler chickens.

Groups	Time (dpi)									

	0^+^	7	14*	21	28^#^	35	35CH	42	42CH	Total
A1	4	4	4	4	4	4	4	4	4	36
B1		4	4	4	4	4	4	4	4	32
B2				4	4	4	4	4	4	24
B3				4	4	4	4	4	4	24
Total										116

Each chicken in Group B was inoculated with the UPM08136P20B1 at day 0 of the trial (+). Booster doses were administered to chickens in the booster groups at 14 dpi (*). Pathogenic FAdV 8b virus was used to infect chickens in the challenge groups at 28 dpi (#). Four chickens in each group were culled on each sampling day as indicated in the table. dpi=Day post-inoculation

### Experimental data collection, presentation, and statistical analysis

#### Gross lesions and histological changes

Liver, spleen, and thymus samples from each chicken in this experiment were examined for gross lesions and fixed in 10% buffered formalin for 48 h [19], processed, hematoxylin and eosin (HE) stained, and examined under a simple light microscope (Leica DM LB2) to assess histopathological changes as previously described [3]. Images were captured (Leica DFC295) and recorded.

#### Serum sample analysis using an enzyme-linked immunosorbent assay (ELISA)

Serum was harvested from each blood sample collected within 24 h, centrifuged at 240× g for 5 min, and stored at –20°C until use. Samples from four chickens were tested for maternal antibodies on day 0 of the experimental trial. This test was performed at the Laboratory of Vaccines and Therapeutics, Institute of Bioscience, UPM. An ELISA kit (BioChek, UK) was used according to the manufacturer’s instructions and was read at 405 nm using an ELISA reader (Dynatech MR7000, USA).

### Flow cytometric immunophenotyping analysis

Samples from chickens in each group on each sample day were softly macerated, filtered through a 70-µm cell strainer (FALCON-Corning, NC, USA) into a centrifuge tube, and centrifuged at 352× g for 5 min. The cell pellets were suspended in 1 mL of PBS and counted; cells equivalent to 1 × 10^6^/mL from each sample were transferred to a Falcon tube (FALCON-Corning) and stained with Mouse Anti-Chicken CD3-FITC (Fluorescein isothiocyanate) [20], Mouse Anti-Chicken CD4-APC (Allophycocyanin) [21], and Mouse Anti-Chicken CD8-PE (Phycoerythrin) [20] antibodies (SouthernBiotech, Birmingham, AL, USA). The cells were then washed with phosphate-buffered saline (PBS) (PH7.4, 0.01 M, 4°C) 3× and suspended in 500 L of PBS for CD3+, CD4+, and CD8+ phenotyping using a BD FACS (Becton, Dickinson fluorescence-activated cell sorting) Calibur flow cytometer (BD Biotec., San Diego, CA, USA). The generated data were analyzed using Cell Quest software (BD Biotec.).

### Viral genome copy number in the liver and cloaca of challenged chickens with FAdV challenge virus

Primers qHex-F 5′-GTTAGACACCACCG CACAGA-3′/qHex-R 5′-GTCACGGAA CCCG ATGTAGT-3′ and probe qHex Probe 5′-FAM/CCCTC CTTCTGAGTACGGAGAG-3′ BHQ1 for FAdV quantitative polymerase chain reaction (qPCR) (Microgen, Surrey, UK) designed based on partial sequence of the hexon gene of the UPM11142CELP3EP2 challenge virus were used to obtain the standard cure and detect FAdV. The FAdV-positive control with an initial DNA concentration of 100 ng/µL, which was diluted seven-fold from 100 ng/µL to 0.0001 ng/µL, was used to generate the standard curve. These dilutions were amplified in triplicate to generate an amplification plot and standard curve with an efficiency of 96%, R square of 0.997, slope value of 3420, and Y-intercept of 26.008. The total volume of the qPCR reaction mix was 20 µL containing 10 µL of SensiFAST™ probe no-ROX kit (Bioline, London, UK), 0.8 µL of primer pair, 0.2 µL of probe, 4.2 µL of nuclease-free water, and 4 µL of template. The non-template control was added in triplicate, and nuclease-free water was used as the template. qPCR amplification was performed using the CFX96™ real-time PCR Detection System (Bio-Rad, USA). The qPCR conditions were as follows: initial denaturation at 95°C for 2 min and 40 cycles of denaturation and extension at 95°C for 5 s and 60°C for 20 s, respectively. CTs of all replicates were obtained, the mean of each sample was determined, and the copy number was calculated.

### Statistical analyses

Two-way repeated-measures analysis of variance on Statistical Package for the Social Sciences (SPSS) 25.0 for Windows (SPSS Inc., Chicago, IL, USA) was used to analyze the differences within and between groups with a 5% probability using the *post hoc* Tukey HSD test [18]. A probability value of p < 0.05 was considered significant. The results generated in this study are presented in Tables and Figures.

## Results

### Attenuation of FAdV 8b isolate UPM08136P5 expression in CEL cells

**Figure-1:**
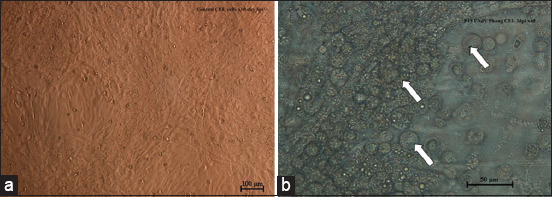
Inverted microscopic images of CEL cells monolayer with cytopathic effects after infection with fowl adenovirus 8b isolate. (a) Confluent CEL cells uninfected control; (b) CEL cells infected with UPM08136P5 exhibiting CPE clumping, rounding, and detachment of cells from the monolayer (10 ×; b 20 ×). CEL=Chicken embryo liver, CPE=Cytopathic effect.

Zero CPE was observed on day 1 of P1, but 80% CPE was observed on day 3 of P1 (Table-S1). The observed CPE included clumping, rounding, and cell detachment from the monolayer (Figure-1). The P5 isolate showed 80% CPE at 3 dpi, P15 at 4 dpi, and P19 at 6 dpi, indicating delayed CPEs.

**Table-S1:** Percentage CPE of UPM08136 passages on CEL Cells post inoculation.

Passages	Day post inoculation					

	1	2	3	4	5	6
1	No CPE (0/3)	80% CPE (3/3)				
2	20% CPE (2/3)	80% CPE (3/3)				
3	No CPE (0/3)	No CPE (0/3)	50% CPE (3/3)	80% CPE (3/3)		
4	No CPE (0/3)	80% CPE (3/3)				
5	No CPE (0/3)	50% CPE (3/3)	80% CPE (3/3)			
6	30% CPE (3/3)	80% CPE (3/3)				
7	30% CPE (3/3)	80% CPE (3/3)				
8	CLUMPING (3/3)	80% CPE (3/3)				
9	40% CPE (3/3)	80% CPE (3/3)				
10	CLUMPING (1/3)	60% CPE (3/3)	80% CPE (3/3)			
11	NO CPE (0/3)	NO CPE (0/3)	80% CPE (3/3)			
12	NO CPE (0/3)	NO CPE (0/3)	80% CPE (3/3)			
13	10% CPE (1/3)	70% CPE (3/3)				
14	NO CPE (3/3)	70% CPE (3/3)	80% CPE (3/3)			
15	NO CPE (0/3)	NO CPE (0/3)	40% CPE (2/3)	80% CPE (3/3)		
16	NO CPE (0/3)	CLUMPING (3/3)	80% CPE (3/3)			
17	CLUMPING (2/3)	50% CPE (2/3)	80% CPE (3/3)			
18	NO CPE (0/3)	CLUMPING (1/3)	40% CPE (2/3)	80% CPE (3/3)		
19	NO CPE (0/3)	NO CPE (0/3)	CLUMPING (1/3)	30% CPE (2/3)	60% CPE (3/3)	80% CPE (3/3)
20	CLUMPING (2/3)	60% CPE (3/3)	70% CPE (3/3)	90% CPE (3/3)		

### Sequence alignment and analysis of the hexon and fiber genes

**Figure-2:**
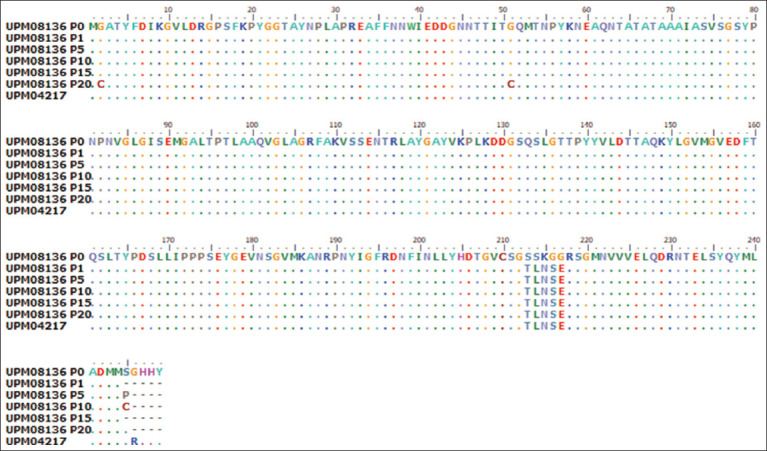
Amino acid residues of the hexon gene of passage isolates and reference isolate from GenBindicateting changes between the initial and attenuated isolate.

The PCR products of the hexon gene were 738–750 bp in length with coding nucleotides in length, which had 98%–100% homology with the UPM04217 reference strain and other FAdV 8b isolates in GenBank and encoded 246–250 amino acid sequences. The hexon gene alignment results revealed numerous synonymous and non-synonymous nucleotide changes. There were G
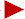
4T and G
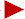
151T nucleotide changes that occurred between passages 0 and 20 which encoded G
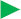
2C and G
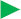
51C changes in the amino acid sequence. We observed a unique SSKGG substitution at position 213–217 of UPM08136EEP1, which differed from TLNSE in other passage isolates and strains from GenBank (Figure-2) and was encoded by 8 nucleotide changes.

**Figure-3:**
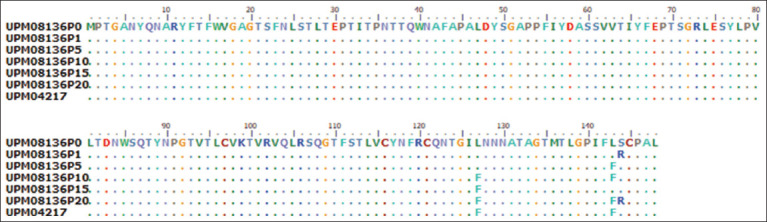
Amino acid residues of the fibe gene of passage isolates and reference isolate from GenBindicateting changes between the initial and attenuated isolate.

**Figure-4:**
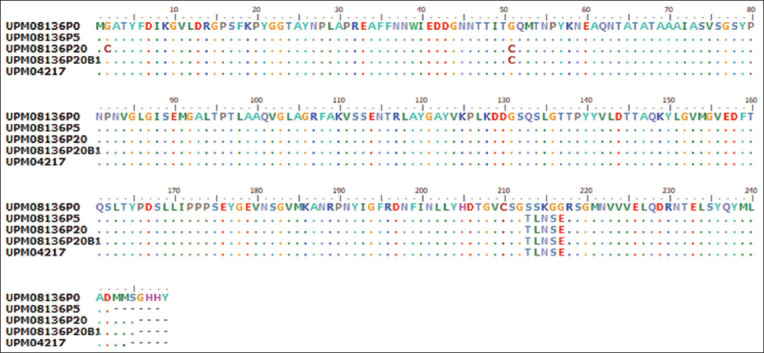
Comparison between the amino acid sequences of the hexon gene of the flask and bioreactor isolates indicating one amino acid change.

**Figure-5:**
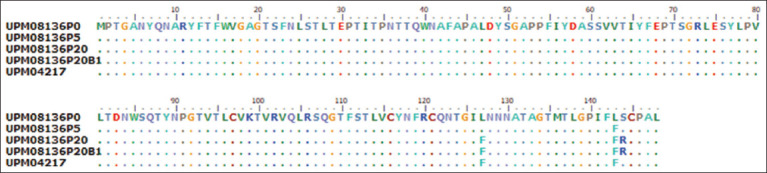
Comparison between the amino acid sequences of the fiber gene of the flask and bioreactor isolates indicating one amino acid change.

The amplicons of the UPM08136 Fiber gene had 430–500 bp amino acid encoding nucleotides, which were 98.91% homologous with the UPM04217 reference strain and other FAdV 8b isolates in GenBank and encoded approximately 136 amino acid sequences. The alignment results of the fiber gene of UPM08136 P0-P20 isolates showed nucleotide changes A
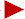
177G, G
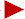
429T, A
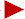
430C and C
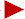
432T between P0 and P20 which resulted in L
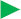
127F, L,
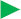
143F and S
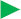
144R amino acid changes between P0 and P20 (Figure-3). The alignment of the amino acid sequences of hexon genes (Figure-4) of UPM08136P20 and UPM08136P20B1 showed that there was a non-synonymous G
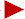
4T change in UPM08136P20B1 compared with that in UPM08136P20, but that of the fiber gene (Figure-5) showed no changes. The DNA sequences of the isolates were uploaded to GenBank (National Center for Biotechnology Information [NCBI]) and accession numbers were obtained (Table-2).

**Table-2:** National Center for Biotechnology Information GenBank accession numbers of passage isolates in this study.

S. No.	Isolates	Accession numbers	

		Hexon gene	Fiber gene
1	UPM08136EEP1	MT127101	MT479176
2	UPM08136CELP1	MT212042	MT525006
3	UPM08136CELP5	MT212045	MT525009
4	UPM08136CELP10	MT212048	MT525012
5	UPM08136CELP15	MT212051	MT525015
6	UPM08136CELP20	MT212054	MT525018
7	UPM08136CELP20B1	MT561445	MT561449

### Clinical signs, gross lesions, and histopathological changes

**Figure-6:**
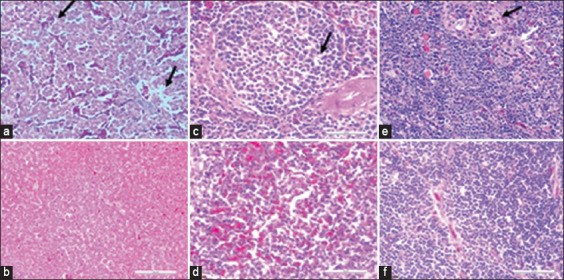
Liver, spleen, and thymus of challenged and unchallenged chickens inoculated with attenuated FAdV 8b UPM08136P20B1: (a) liver of challenged control chickens showing necrosis, congestion, and vacuolation of the hepatocytes (black arrows). (b) liver of unchallenged control chickens. (c) spleen of challenged control chickens showing cellular vacuolation and necrosis (black arrow). (d) spleen of unchallenged control chickens. (e) thymus of challenged control chickens showing lymphoid cells depletion (black and white arrows). (f) unchallenged control respectively. (b, d, and f) thymus of unchallenged chickens showing normal conformity, at 35 dpi. Hematoxylin and eosin, 40 ×. dpi=Day post-inoculation, FAdV=Fowl adenovirus.

Depression and inappetence within 2-day post-challenge were observed in two chickens: A1-challenged chickens had pale, discolored, and enlarged liver (two chickens), splenomegaly (one chicken), and enlargement of the thymus (four chickens), which were not observed among the unchallenged A1 and challenged B groups. There were necrosis, congestion, and vacuolation in the liver (Figure-6a), congestion, vacuoles, and nuclear debris in the spleen (Figure-6c), reduced cortex thickness, cell depletion in the medulla, and signs of lymphoid depletion in the thymus (Figure-6e) of challenged chickens in the A1 group, which were absent among chickens in the unchallenged chickens in the A1 group and challenged chickens in all the B groups (Figures-6b, d, f).

### Body weight (BW), liver weight (LW), and liver: Body weight ratio (LBR)

**Figure-7:**
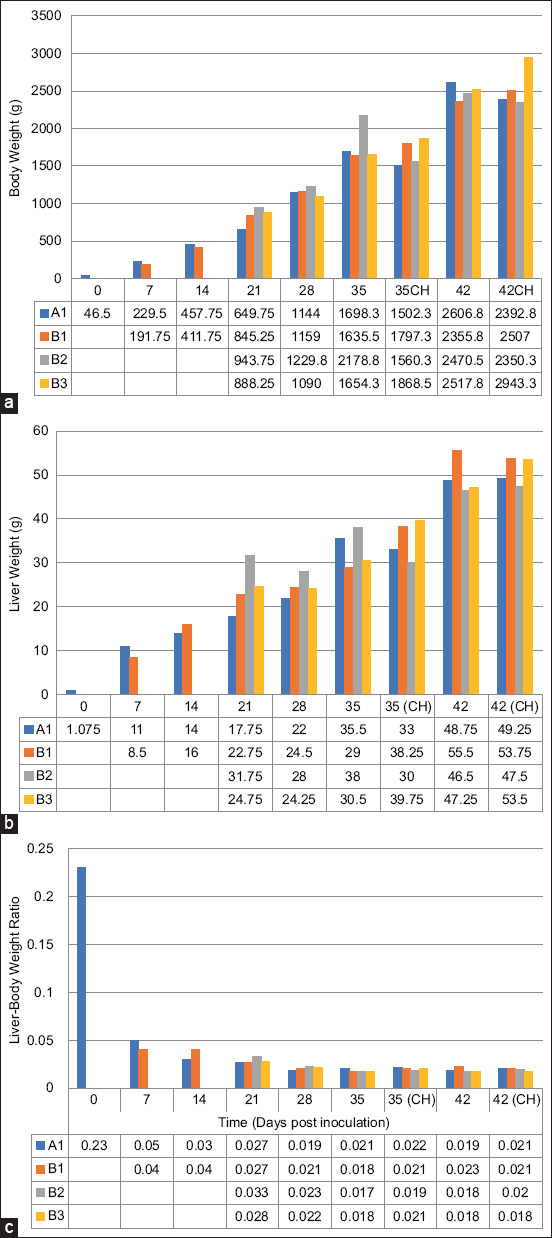
(a) body weight, (b) liver weight, and (c) body-to-liver-weight ratio, respectively, of commercial chickens inoculated with attenuated UPM08136P20B1 FAdV 8b strain. A1=Uninoculated control; B1=Inoculated without booster; B2=Inoculated, + attenuated booster; B3=Inoculated, + booster killed; CH=Challenged. FAdV=Fowl adenovirus.

BW was significantly higher (p = 0.05) at 21 and 35 (A2 only) dpi in the inoculated chickens (A group) than that in the uninoculated control group but was similar throughout the trial (Figure-7a). Similarly, BW was significantly higher (p < 0.05) at 35 and 42 dpi in the inoculated chickens than in the uninoculated control chickens. Among the inoculated chickens, chickens in the B3CH group exhibited significantly higher (p < 0.05) BW. At 7 dpi, the LW of the chickens in the inoculated group was lower (p > 0.05) than that of the uninoculated group, but the values remained statistically similar throughout the rest of the trial (Figure-7b). The LBR of challenged control chickens was higher than that of inoculated chickens at 35 and 42 dpi (Figure-7c).

### FAdV antibody

**Figure-8:**
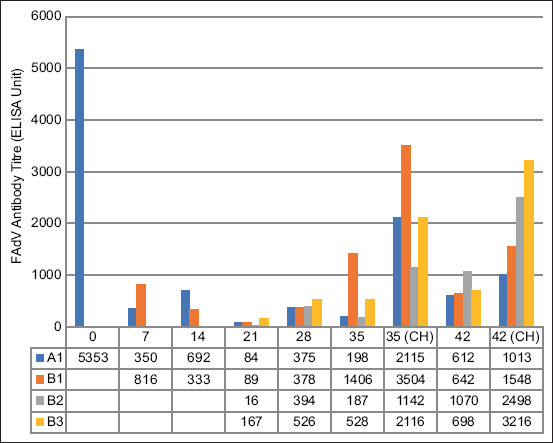
FAdV antibody titer of commercial chickens inoculated with attenuated UPM08136P20B1 FAdV 8b strain. A1=Uninoculated control; B1=Inoculated without booster; B2=Inoculated, + attenuated booster; B3=Inoculated, + booster killed; CH=Challenged. FAdV=Fowl adenovirus.

Unchallenged chickens in the A1 group exhibited an antibody titer of 5353 ± 769 at 0 dpi, which declined significantly throughout the trial, indicating high maternal antibodies (Figure-8). A significantly higher (p < 0.05) titer was recorded at 35 dpi in the non-booster group (B1), whereas a significantly higher (p < 0.05) titer was recorded at 42 dpi in the booster with attenuated virus group (B2) compared with all the groups. At 35 and 42 dpi, the challenged chickens in the non-booster and booster with inactivated virus groups showed higher titers than the other groups.

### CD3+, CD4+, and CD8+ T lymphocyte subpopulations in the liver, spleen, and thymus

**Figure-9:**
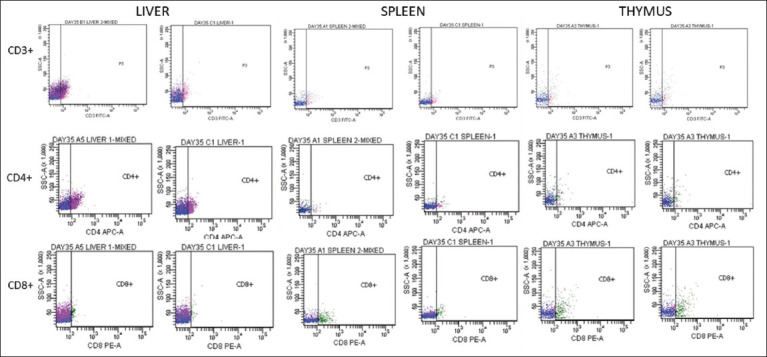
Flowcytometric dot-plot of CD3+, CD4+, and CD8+ in the liver, spleen, and thymus of challenged chickens inoculated with attenuated UPM08136P20B1at 35-day post-inoculation (7-day post-challenge).

Upregulation of CD3+, CD4+, and CD8+ T lymphocytes was observed in the organs, as shown in the dotted plots (Figure-9). At 14, 21, 28, 35, and 42 dpi, the percentage of CD3+ T lymphocytes in the liver of chickens inoculated with UPM08136P20B1 was higher than that of the uninoculated control group (p > 0.05). The spleen had a higher mean value at 14, 21, and 35 dpi, whereas the thymus had a higher mean value at 7, 14, 28, and 42 dpi. At 7, 2, 1, 28, and 42 dpi, the percentage of CD4+ T lymphocytes in the liver of chickens inoculated with UPM08136P20B1 was higher (p > 0.05) than that of the uninoculated control group. In the spleen, it was higher at 14 and 21 dpi, whereas in the thymus, it was higher at 14 and 28 dpi. This indicates the excitement of cellular immunity because of inoculation with attenuated FAdV. The number of CD8+ T lymphocytes was higher (p > 0.05) in the liver of inoculated chickens at 14, 21, 28, 35, and 42 dpi, in the spleen at 14, 21, and 35 dpi, and in the thymus at 14 and 28 dpi. This indicates that attenuated FadV inoculation induces cytotoxic cellular immunity.

The percentages of CD3+ and CD4+ T lymphocytes were higher in the liver and spleen at 35 and 42 dpi and in the thymus at 42 dpi among the challenged chickens. The CD3+ and CD4+ T-cell subpopulations were similar at different time points for the challenged and unchallenged chickens in the inoculated groups; however, a significantly higher percentage (p < 0.05) was recorded in the liver and thymus of the non-booster group at 35 dpi. The booster with inactivated FAdV group had a significantly higher percentage in the spleen at 35 dpi, whereas the booster with attenuated FAdV group had a significantly higher percentage in the thymus at 21 and 42 (challenged) dpi. The percentages of CD8+ T lymphocytes were higher in the liver at 35 and 42 dpi and in the thymus at 42 dpi.

### Viral load and shedding in the liver

The FAdV challenge virus genome copy number at 35 and 42 dpi was significantly higher (p > 0.05) in the livers of chickens in the A1 challenged group than that of chickens in the non-booster group and higher (p > 0.05) than that of chickens in the booster groups (Table-3), indicating reduced virus load among the inoculated chickens. At 35 dpi, the copy number of challenge FAdV 8b in the cloaca of challenged control chickens in the A1 challenged group was significantly higher than that of the chickens in all the inoculated booster groups, was significantly higher than that of the chickens in the non-booster group, and was significantly higher (p > 0.05) than that of the booster with attenuated FAdV group, indicating reduced virus shedding among the inoculated chickens.

**Table-3:** Copy number of FAdV challenge virus in the liver and cloaca of chickens inoculated with attenuated FAdV strain (UPM08136P20B1) and challenged.

Group	35-dpi (7-day post-challenge)	42-dpi (7-day post-challenge)
Liver		
A1	7.71 ± 0.07^b^	8.03 ± 0.11^b^
B1	7.19 ± 0.09^a^	7.26 ± 0.07^a^
B2	7.42 ± 0.05^a,b^	7.83 ± 0.04^b^
B3	7.38 ± 0.09^a,b^	7.68 ± 0.03^b^
Cloaca		
A1	8.54 ± 0.01^c^	8.17 ± 0.04^b,c^
B1	7.79 ± 0.02^a^	7.74 ± 0.03^a^
B2	7.69 ± 0.06^a^	8.02 ± 0.02^b^
B3	8.10 ± 0.09^b^	8.22 ± 0.02^c^

A1=Uninoculated control; B1=UPM08136 inoculated, non-booster; B2=UPM08136 inoculated, booster Live; B3=UPM08136 inoculated, booster killed groups. ^a,b,c^superscripts that are different are significantly different (p < 0.05) in the same column while the same superscripts are not significantly different (p > 0.05). dpi=Day post-inoculation, FAdV=Fowl adenovirus

## Discussion

In this study, the FAdV serotype 8b isolate UPM08136P20B1 was attenuated in flask CEL cell culture with novel molecular changes in the hexon and fiber genes and propagated in a stirred tank bioreactor without effect. The attenuated isolate-induced humoral and cellular immunity that protected commercial broiler chickens was challenged with pathogenic FAdV 8b and reduced viral load in the liver and cloaca.

Attenuation of viruses involves serial passage in susceptible host cells. Typical CPEs were observed with UPM08136 passage isolates on CEL cells, similar to FAdV 4 on CEL cells [15]; however, delayed CPEs started occurring from P14 to P15, which could indicate that the infectivity of the isolate is reducing, an indication of probable attenuation. Reduced pathogenicity and retained immunogenicity of viruses are expected through serial passages [22]. CPE within 2 days translating to 6 days could indicate attenuation; however, such a presumption could only be confirmatory when backed with molecular changes [23].

Because viruses attempt to adapt to new hosts, such as animals, plants, embryonated eggs, or cell culture, genetic changes usually occur that alter the virus pathogenicity, leading to attenuation [24]. Substitutions in hypervariable FAdV regions, such as the L1 loop region of the hexon gene or the knob region of the fiber gene, may lead to attenuation. This can be exhibited as delayed CPEs such as UPM08136P20 on CEL cells, and such changes would be markers of attenuation. In FAdV 8b, the L1 loop region of the hexon gene is a hypervariable region where most changes that lead to homology occur [25]. Because the antigenic binding activity of FAdV occurs most often in the L1 loop region [26], changes that transpire here could be markers of virus infectivity and attenuation [12]. Fiber genes also play an important role in the pathogenicity of FAdV because they are the first to be exposed to the environment, making it prone to early molecular changes [25]. In addition, these changes in FAdV fibers alone can alter their pathogenicity [27]. Unfortunately, there is no consensus on the exact marker for FAdV 8b attenuation.

A change from glycine, a non-polar amino acid, to cysteine, a polar amino acid, observed in UPM08136P20 could affect the structure of the hexon protein and alter the functionality or pathogenicity of the virus. *Staphylococcus epidermidis* has been reported to exhibit a change similar to a variant with the ability to tolerate temperatures ranging from 25°C to 50°C [28]. In Type 1 collagen, a guanine-cytosine (GC) substitution changes the conformity of the triple helix [29]. The TLNSE motif is unique to UPM08136P0 because it was not found in its progeny or in our reference strain. The change to SSKGG, which coincides with the change from low to high pathogenicity in SPF embryonated egg and CEL cells, could be linked to the adaptation of the virus to CEL cells and/or its pathogenicity. In addition, a change in leucine to phenylalanine in the knob region of the fiber could also be a marker for delayed CPE and thereby attenuation of this FAdV isolate. A similar change from leucine to phenylalanine in the Japanese encephalitis virus caused a hindrance in the assembly of the virus particles but did not affect endocytosis, which led to viral attenuation [30]. It is probable that this change may be responsible for the observed delayed CPE, and it has been proposed to be an attenuation marker of UPM08136. Other changes in other genes could be determined by multiple gene sequencing or whole-genome sequencing; however, the hexon and fiber genes are the major pathogenic genes of FAdV 8b [25, 31]. Therefore, further studies, which may include whole-genome sequencing, are warranted to fully show the changes in other regions and genes, especially during field trials to monitor the virus. Interestingly, UPM08136P20B1 was stable after bioreactor propagation, which is exemplified by no changes in the studied genes, similar to previous reports of viruses propagated in bioreactors [32–34].

The UPM08136P20B1 isolate inoculated into broiler chickens did not cause mortality throughout the trial, similar to a previous report [35]. However, there were minimal clinical signs, gross lesions, and histopathological changes among chickens in the uninoculated challenged group, similar to those reported by other researchers [36, 37]. However, the lack of these signs, lesions, and changes among chickens in the non-challenged and inoculated challenged groups shows that attenuated FAdV 8b is safe and effective.

The results of the BW and LW tests indicated the safety and protective efficacy of attenuated FAdV 8b. The higher LBR among the uninoculated control-challenged (UCC) group showed protection of the liver from inoculated chickens, which indicated the protective efficacy of the attenuated FAdV 8b because splenomegaly is a major lesion of FAdV 8b infection in chickens [38].

High maternal antibody levels in chickens declined to 21 dpi, which may have affected antibody induction by UPM08136P20B1 attenuation. Maternal antibodies from breeder chickens to their chicks provide protection in the early life of the chicks [39], but they are short-lived and can aid in the suppression of the chick’s complete response to vaccination early in life [40, 41], mostly with attenuated vaccines that require infection to induce protection. Maternal antibodies may suppress immunity by blocking B&T helper cell release by activating T suppressor cells and inhibiting antigen processing [42]. Physical epitope blocking, a process referred to as epitope masking [43, 44], can also be performed. Maternal antibodies suppress the immune response to other viruses, such as infectious bursal disease virus [45] and Newcastle disease virus [46]. Induction of passive immunity by FAdV has also been reported [47, 48]. However, UPM08136P20B1-induced antibodies were higher than those of uninoculated control chickens at different time points, especially after the decline of maternal antibodies. Interestingly, among the challenged chickens, the B1 group with the lowest antibody titer at day 42 pi also had the least viral load in the liver at 42 dpi, suggesting active virus neutralization, a sign of protective efficacy. However, it is possible that other protective pathways may be involved following the report of FAdV 8b vaccines providing protection to chickens after challenge even with low antibody titers [13].

Attenuated FAdV 8b-induced CD3+, CD4+, and CD8+ T-lymphocytes in the liver, spleen, and thymus of broiler chickens in this study. The efficacy of viral vaccines has been correlated with their ability to induce cell-mediated immune responses [12]. Live-attenuated vaccines mimic natural infections and should induce cellular immunity [49]. High levels of CD3 T cells have been reported among chickens inoculated with FAdV 8 up to 25 dpi [50], but CD3+, CD4+, and CD8+ cells were induced up to 42 dpi in this trial. These findings were more remarkable in the liver, which was the primary organ of infection at day 35 pi, but also in the spleen and thymus at different time points. The spleen is an important organ in the host’s immune response to viral infections because it interacts with the blood and pathogens. Therefore, the upregulation of lymphocytes in the spleen in this trial is an indication of the induction of memory cells, which is necessary for long-term vaccine efficacy [51].

The thymus, where T cells mature, plays an important role in the development of cellular immunity [50]. However, FAdV 4 reduced the percentage of CD4+ and CD8+ cells in the thymus [13]. This is similar to the 86% and 83% decrease in CD8+ cells in the thymus and liver, respectively, at 35 dpi among the uninoculated challenged control group and from 19.70 ± 3.55 to 10.77 ± 2.16 (45% reduction) among chickens in the A1 group. Therefore, attenuated FAdV 8b would protect inoculated chickens from lymphoid depletion and lymphocyte apoptosis.

Attenuated FAdV 8b significantly reduced the shedding of the challenge virus, indicating that blocking immunity was induced. Considering the effect of horizontal transmission on the epidemiology of FAdV 8b infection, this finding is remarkable. The ability to reduce or stop shedding should be a measure of the efficacy of vaccines [52] because it is a way of eradicating disease.

Overall, chickens in the inactivated booster group had better BW and LBR, whereas those in the non-booster group had higher antibody and virus shedding results, whereas chickens in the attenuated booster group had better CMI results. Thus, it can be deduced that each regimen could protect chickens against FAdV 8b challenge; however, the booster with inactivated inoculation appeared to be the best.

## Conclusion

The UPM08136P20B1 isolate was attenuated in CEL cell flask culture and adapted to bioreactor propagation. The isolate was immunogenic and protected broiler chickens against pathogenic FAdV 8b. Chickens in the inoculated groups were protected from histopathological changes in the studied organs and had higher BW, lower LBR, and higher T-lymphocyte subpopulations in the liver, spleen, and thymus. The inoculated chickens had significantly lower FAdV copy number in the liver and cloaca than the control group chickens. UPM08136P20B1 could be used as a vaccine to prevent and control IBH caused by FAdV serotype 8b.

## Data Availability

The supplementary data can be available from the corresponding author on a reasonable request. The DNA sequences of the isolates were deposited into the NCBI GenBank and the accession numbers are provided in Table-1.

## Authors’ Contributions

MHB, ARO, and AI: Conceptualized the study. CCU: Conducted the experiments, laboratory works, data collection, and wrote the original draft. CCU, MIN, and ARO: Performed data and molecular analysis. MHB, ARO, MIN, and AI: Supervised the study. CCU, MHB, ARO, MIN, and AI: Reviewed and edited the manuscript. All authors have read and approved the final manuscript.
